# Analysis of headache burden Chinese in the global context from 1990 to 2021

**DOI:** 10.3389/fneur.2025.1559028

**Published:** 2025-04-16

**Authors:** Wenxuan Zhao, Yonglin Chen, Yushi Tang, Shiqing Du, Xiaojie Lu

**Affiliations:** ^1^Department of Neurosurgery, Jiangnan University Medical Center, Jiangnan University, Wuxi, China; ^2^Wuxi Neurosurgical Institute, Wuxi, Jiangsu Province, China

**Keywords:** headache, migraine, tension-type headache, joinpoint, APC model

## Abstract

**Background:**

Headache is one of the leading causes of disability in worldwide and in China, and the second most burden disease of the nervous system after stroke. We aimed to analyze the global trends in the burden of headache disorders, migraine and TTH among people in China in an international context.

**Methods:**

Data were taken from the Global Burden of Disease (GBD) Study 2021 database to examine trends in the incidence, prevalence, and disability-adjusted life year of headache disorders.

**Results:**

In the context of a large scale in the world, headache is currently in a state of high incidence in China, and female middle-aged and young patients are the main body of this disease.

**Conclusions:**

Need to focus on the vulnerable population in China, while formulating effective health policies and developing good treatment measures to reduce the incidence of headache disorders and improve the DALYs rate of headache.

## 1 Introduction

Headache is the second most common neurological disease in adults after stroke (1). Among them, primary headache is the most common type of headache (2–4), which has caused a heavy burden to the world and national economies. The common types of headaches are migraine and tension-type headache (TTH). Migraine is a recurrent disorder characterized by unilateral, moderate to severe throb headache (5), accompanied by other constitutional symptoms such as photophobia (6), phonophobia, abnormal skin pain (7), and gastrointestinal disorders (8). Migraine affects an estimated 1 billion people worldwide (9). TTH is usually less painful than migraine, is predominantly bilateral, oppressive, or tight, and is not exacerbated by daily activities. Relief can be achieved by various methods, such as getting enough sleep, taking appropriate medications and massage. Despite the high incidence and potential disability of TTH, little progress has been made in this field due to a lack of attention and resource investment from the scientific community, funders, and the pharmaceutical industry since the early 2000s (10). Headache has become a key factor affecting the quality of life of adults, and with the increase of life pressure, the population affected by headache tends to be younger. Research, education, and clinical services for migraine and tension-type headache are scarce in developing countries. Even in economically developed regions, many people with migraine and TTH receive inadequate or no treatment at all.

Up to now, there is a lack of epidemiological studies on headache in China. To fill this gap, we analyzed the data of headache disorders in people from the Global Burden of Disease Database from 1990 to 2021, to explore the trends in incidence, prevalence, and disability-adjusted life years (DALYs), and to predict the future trend of disease burden, Joinpoint regression Model, Age-Period-Cohort (APC) Model and Autoregressive Integrated Moving Average (ARIMA) were used.

The aim of this study is to provide policy makers with insights into the overall burden of headache disorders in China, assist in the development of precise prevention strategies, and realize the rational allocation of public health resources.

## 2 Methods

### 2.1 Data source

The data is sourced from GBD 2021 (http://ghdx.healthdata.org/gbd-results-tool). This study encompasses 204 countries/regions, 369 diseases and injuries, as well as 87 risk factors. It integrates multiple information sources such as census, surveys, vital statistics, and health databases (11, 12). Based on the GBD database, we screened the crude incidence, crude prevalence, and crude DALYs (Disability-Adjusted Life Years) rates of cephalic pain (including migraine and tension headache) in China and globally from 1990 to 2021. Additionally, we screened the age-standardized incidence, prevalence, and DALYs rates to measure the burden of cephalic pain.

### 2.2 Research method

#### 2.2.1 Time-trend analysis

Using the Joinpoint regression model, we calculated statistically significant tipping points to show how migraine incidence changed in China from 1990 to 2021. We verified this trend using APC (annual percentage change) for each period and AAPC (average annual percentage change) for the whole study period.

#### 2.2.2 Age-period-cohort (APC) model analysis

The Age-period-cohort (APC) model, widely used in epidemiological research, analyzes health trends like chronic disease incidence by breaking down data into age, period, and cohort dimensions. Its formula is ln (Rapc) = μ + α*a* + β*p* + γ*c*, where ln(Rapc) is the incidence rate's natural logarithm, μ is the intercept, α*a* is the age effect, β*p* is the period effect, and γ*c* is the cohort effect. This study uses the Intrinsic Estimator method to avoid bias due to their linear correlation and divides age into 19 groups (5-95+) and period into 6 segments (1992–2021), each spanning 5 years.

#### 2.2.3 Autoregressive integrated moving average (ARIMA) model

The Autoregressive Integrated Moving Average (ARIMA) model combines autoregressive (AR) and moving average (MA) components, represented as ARIMA (*p, d, q*). It transforms non-stationary time series data to stationary, then establishes a regression model using lagged values of the dependent variable and random error term. By viewing time series data as a random sequence and capturing its autocorrelation, ARIMA leverages past-current relationships for accurate future predictions, showcasing its potential in time series forecasting (13).

## 3 Statistical analysis

We screened data from the GBD database for incidence, prevalence, DALYs, and corresponding age-standardized incidence rates (ASIR), age-standardized prevalence rates (ASPR), and age-standardized DALYs rates (ASDR) of headache disorders (HD), including migraine and TTH, in mainland China and globally. We also obtained crude rates by age group. Using Joinpoint software, we calculated the average annual percent change (AAPC) and its 95% confidence interval (95% CI) to assess disease burden trends. Trends were determined by the 95% CI of the AAPC: >0 for increasing, <0 for decreasing, and including 0 for stable. Statistical analysis and data visualization were conducted using R (version 4.1.3) and Joinpoint software (version 4.9.1.0). Statistical significance was set at *p* < 0.05.

## 4 Results

### 4.1 Global and china overall headache trends

We conducted a thorough analysis of headache disease cases, ASIR, ASPR, and ASDR in China and globally and calculated the corresponding annual average percentage changes (AAPC) (Table 1). Over the past three decades, China has witnessed a significant increase in the ASIR for headache diseases, rising from 7,384.8 (95%CI: 6,533.7–8,243.8) in 1990 to 7,826.7 (95%CI: 6,910.7–8,716.5) in 2021. The AAPC for this period was 0.1930 (95%CI: 0.1537–0.2324), *p-*value < 0.001. Similarly, ASPR also showed an upward trend, increasing from 25,797.5 (95%CI: 23,744.2–27,933.3) to 27,582.6 (95%CI: 25,492.6–29,985.8), with an AAPC of 0.2208 (95%CI: 0.1793–0.2623) (*p* < 0.001). Furthermore, ASDR also increased, from 454.8 (95%CI: 93.9–960.9) to 487.2 (95.4–1,036.8), with an AAPC of 0.234 (ranging from 0.1867 to 0.2814) (*p* < 0.001). In contrast, the global ASIR for headache diseases exhibited a slight decline, falling from 10,097.2 (95%CI: 8,965.2–11,186.3) in 1990 to 10,084.5 (95%CI: 8,956.5–11,170.8) subsequently, with an AAPC of −0.0009 (95%CI: −0.0095–0.0077). It is evident that China's growth rates in headache disease ASIR, ASPR and ASDR are all above the global average (Table 1).

**Table 1:** Case numbers, age-standardized incidence rate, prevalence rate, DALY rate and corresponding AAPC of headache disorders in China and the world in 1990 and 2021.

**Location**	**Measure**	**1990**		**2021**		**1990-2021 AAPC**	
		**All-age cases**	**Age-standardized rates per 100,000 people**	**All-age cases**	**Age-standardized rates per 100,000 people**	**AAPC(p-value)**	
		***n*** **(95%UI)**	***n*** **(95%CI)**	***n*** **(95%UI)**	***n*** **(95%CI)**	***n*** **(95%CI)**	***p***
**China**	**Incidence**	88,448,504.1 (77,815,263.9, 99,129,998.5)	7,384.8 (6,533.7, 8,243.8)	115,067,562.2 (101,819,216.6, 128,172,075.8)	7,826.7 (6,910.7, 8,716.5)	0.1930(0.1537, 0.2324)	<0.001
	**Prevalence**	309,197,884.3 (283,206,149.2, 337,800,281.3)	25,797.5 (23,744.2, 27,933.3)	426,012,548.0 (392,887,359.8, 461,775,928.4)	27,582.6 (25,492.6, 29,985.8)	0.2208(0.1793, 0.2623)	<0.001
	**DALYs**	5,518,250.1 (1,088,133.6, 11,790,016.8)	454.8 (94.0,960.9)	7,704,363.5 (1,631,692.8, 16,157,988.0)	487.2 (95.4, 1,036.8)	0.234 (0.1867,0.2814)	<0.001
**Global**	**Incidence**	533,794,823.9(472,464,996.9, 591,430,931.6)	10,097.2 (8,965.2, 11,186.3)	809,226,480.2(717,818,771, 895,990,201.5)	10,084.5 (8,956.5, 11,170.8)	−0.0009 (−0.0095, 0.0077)	0.841794
	**Prevalence**	1,787,302,945.1 (1,648,822,201.7, 1,937,346,555.9)	34,486.6 (31,872.8, 37,187.6)	2,808,876,481.8 (2,599,555,367.5, 3,028,767,343.3)	34574.0(32017.7,37318.6)	0.0097(0.0064,0.013)	<0.001
	**DALYs**	30,260,883.9(5,963,392.0, 64,833,434.5)	583.8 (122.5, 1,231.6)	47,975,675.1(9,800,212.3, 100,667,852.5)	588.4 (117.6, 1,245.4)	0.0253(0.0082,0.042)	0.003727

### 4.2 Rough trends in headache disorders and their classification worldwide and in china from 1990 to 2021

We conducted a trend analysis of headache disorders from 1990 to 2021, including their subtypes of migraine and TTH. Although on a global scale, the incidence, prevalence, and DALYs rates of headache disorders, have remained relatively flat over these three decades (Supplementary Figures S1D–F), the number of incidence cases, prevalent cases, and DALYs cases have progressively increased annually (Supplementary Figures S1A–C). Specifically in China (Supplementary Table S1, Supplementary Figures S1G–L), there has been a substantial increase in the number of incident cases of TTH over time, rising from 76,930,406.5 (95%UI: 87,690,508.6–66,380,713.5) in 1990 to 102,020,341.6 (95%UI: 115,000,579.6–88,865,128.1) in 2021. For migraine, the number of incident cases increased from 11,518,097.6 (95%UI: 13,156,841.9–10,091,942.2) in 1990 to 13,047,220.7 (95%UI: 14,698,852.1–11,597,731.5) in 2021. The number of prevalent cases of TTH increased from 204,064,313.2 (95%UI: 233,568,232.2–176,898,604.5) in 1990 to 283,814,151 (95%UI: 320,431,556.8–251,438,661.8) in 2021. Similarly, the number of prevalent cases of migraine rose from 133,474,536.5 (95%UI: 153,482,597.7–114,199,443.7) in 1990 to 184,752,280.1 (95%UI: 213,633,958.3–160,836,524.7) in 2021.

### 4.3 Trends in headache disorders in the world and china from 1990 to 2021 by age-standardization rate

**Figure 1:**
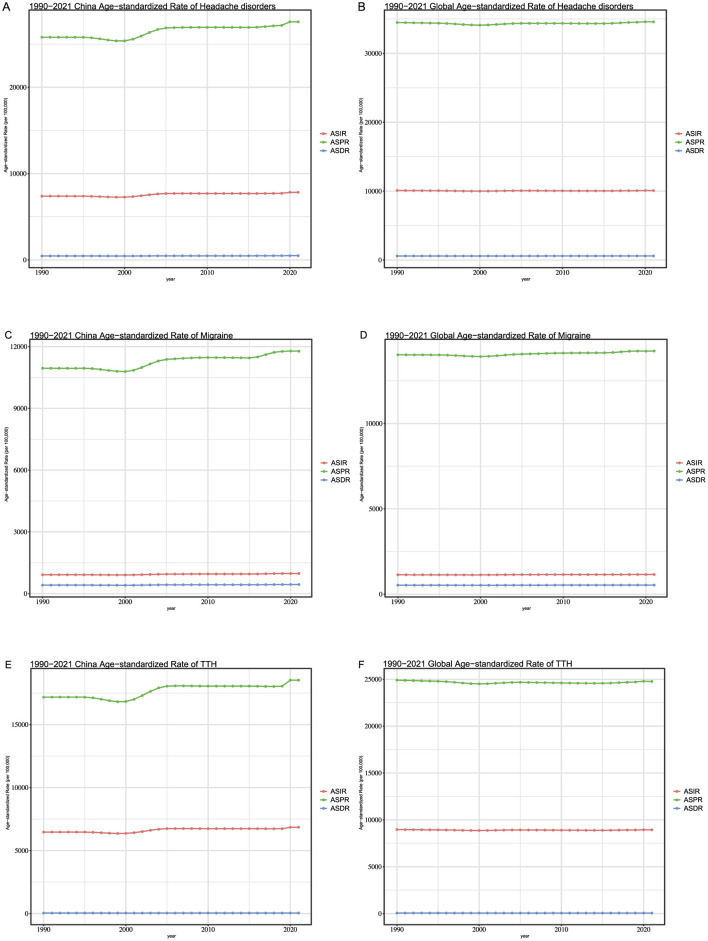
**(A)** The Age-standardized of the incidence, prevalence and DALYs about headache disorders in China. **(B)** The Age-standardized of the incidence, prevalence and DALYs about headache disorders in global. **(C)** The Age-standardized of the incidence, prevalence and DALYs about Migraine in China. **(D)** The Age-standardized of the incidence, prevalence and DALYs about in global. **(E)** The Age-standardized of the incidence, prevalence and DALYs about TTH in China. **(F)** The Age-standardized of the incidence, prevalence and DALYs about TTH in global.

From 1990 to 2021, the ASIR and ASDR for headache disorders in China have remained relatively stable, with no significant fluctuations observed. However, the ASPR showed a slight increase during two specific periods: from 2000 to 2005 and from 2019 to 2021 (Figure 1A). In comparison, on a global scale, these three indicators have similarly maintained stability (Figure 1B). Building on this foundation, we conducted an analysis of the relevant indicators for migraine and TTH. As depicted in the figures (Figures 1C, D), for migraine, both ASIR and ASDR exhibited no significant increase between 1990 and 2021, with a consistently flat trend, a pattern mirrored globally. Regarding ASPR, China experienced two distinct periods of increase, from 2005 to 2010 and from 2015 to 2020, while globally, the trend remained relatively flat. In terms of TTH on a worldwide scale (Figures 1E, F), there were no major variations in ASIR, ASPR, and ASDR overall. In contrast, for China, the ASPR of TTH showed a notable increase. The rate value soared from 17,174.5 (95%CI: 15,086.7–19,380.6) in 1990 to 18,525.1 (95%CI: 16,380.9–20,958.7) in 2021, with particularly significant increases observed during the 2005–2010 and 2019–2021 periods.

### 4.4 Joinpoint regression analysis of the burden of headache disorders in china and the world

**Figure 2:**
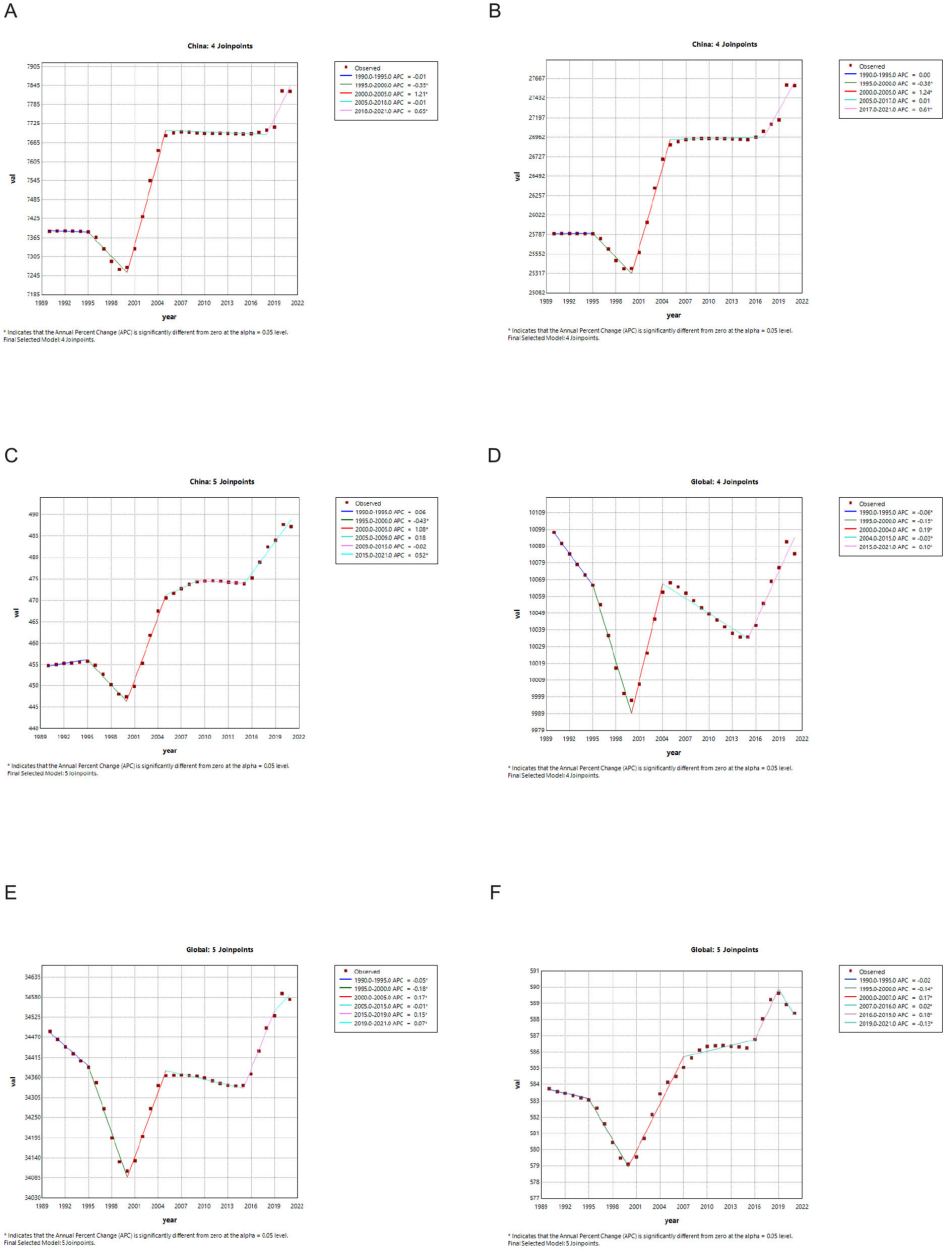
**(A)** Joinpoint regression analysis of the burden of headache disorders of ASIR in China. **(B)** Joinpoint regression analysis of the burden of headache disorders of ASPR in China. **(C)** Joinpoint regression analysis of the burden of headache disorders of ASDR in China. **(D)** Joinpoint regression analysis of the burden of headache disorders of ASIR in global. **(E)** Joinpoint regression analysis of the burden of headache disorders of ASPR in global. **(F)** Joinpoint regression analysis of the burden of headache disorders of ASDR in global.

We analyzed the Joinpoint regression results for the ASIR, ASPR and ASDR of headache in China and globally from 1990 to 2021. In China, the ASIR of headache exhibited a downward trend between 1990 and 2000, particularly from 1995 to 2000 (Annual Percent Change (APC): −0.35, *p* < 0.05) (Figures 2A–C). Between 2000 and 2005, the ASIR increased significantly (APC: 1.21, *p* < 0.05), followed by a slight decline until 2018, but since then, it has increased significantly again from 2018 onwards (APC: 0.65, *p* < 0.05). The trend in ASPR was like that of ASIR, with a decline from 1995 to 2000 (APC: −0.38, *p* < 0.05) and increases during other periods, notably from 2000 to 2005 (APC: 1.24, *p* < 0.05) and from 2017 to 2021 (APC: 0.61, *p* < 0.05). The overall trend in ASDR was upward, especially from 2000 to 2005 (APC: 1.08, *p* < 0.05) and from 2015 to 2021 (APC: 0.52, *p* < 0.05). In contrast, globally (Figures 2D–F), the ASDR has declined significantly since 2019 (APC: −0.13, *p* < 0.05), while the ASIR has increased between 2015 and 2021 (APC: 0.10, *p* < 0.05), and the ASPR has also shown an increasing trend since 2019 (APC: 0.07, *p* < 0.05).

### 4.5 Gender-specific trends in incidence, prevalence, and DALYs in china from 1990 to 2021

We analyzed the trends in incidence, prevalence, and DALYs (Disability-Adjusted Life Years of headache disorders, including migraine and tension-type headache, among different genders in China over the years. The results revealed that across all age groups, women exhibited higher incidence, prevalence, and DALYs for headache disorders compared to men, with this pattern also applying to migraine and tension-type headache. (Supplementary Figure S2) Specifically, in 2021 Chinese data, for migraine, ASIR for women and men were 1,163.4 (95%CI: 1,037.9–1,310.4) and 682.0 (95%CI: 597.1–774.4) respectively, with ASPR was 16,514.4 (95%CI:14,231.2–19,014.6) for women and 9,619.0 (95%CI: 8,330.1–11,169.3) for men. The ASDR was also higher for women, at 610.8 (95%CI: 79.0–1,344.0) compared to 377.0 (95%CI: 79.9–803.7) for men. For TTH, ASIR for women was slightly higher at 7,875.7 (95%CI: 6,894.0–8,888.8) compared to 6,498.0 (95%CI: 5,629.0–7,312.5) for men, with ASPR was 22,113.4 (95%CI: 19,573.5–25,037.0) for women and 17,882.8 (95%CI: 15,603.5–20,356.3) for men. ASDR was also slightly higher for women, at 52.9 (95%CI: 15.6–162.8) compared to 47.9 (95%CI: 15.8–152.9) for men. In summary, the disease burden of both migraine and tension-type headache is greater for women than for men (Supplementary Table S1).

### 4.6 Comparison of the burden of headache, migraine, and TTH in Chinese females and males in 2021

**Figure 3:**
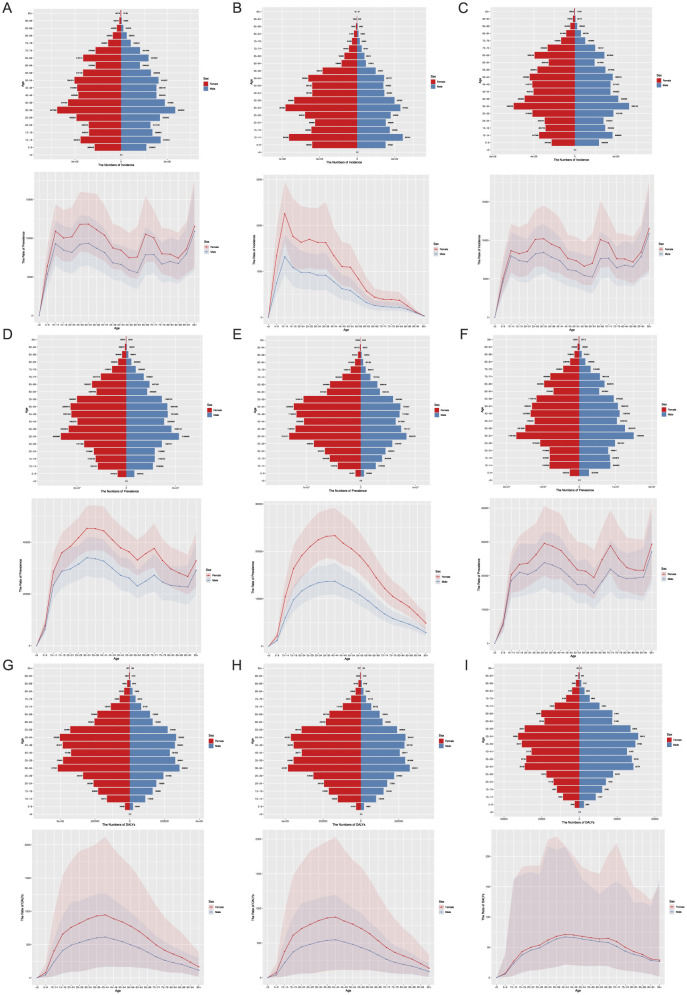
The number and the rate of the incidence of female and male in China in 2021. **(A–C)** The number and rate of incidence about headache disorders, migraine and TTH in female and male in China in 2021. **(D–F)** The number and rate of prevalence about the headache disorders, migraine and TTH in female and male. **(G–I)** The number and rate of DALYs about the headache disorders, migraine and TTH in female and male.

We screened the data on incidence, prevalence, and DALYs for headache disorders in China for the year 2021. We analyzed the number cases and rates by grouping them into 5-year intervals (Figure 3).

#### 4.6.1 Incidence

In the incidence of headache disorders, as illustrated in the figures (Figure 3A), among women within the age range of 10–69, the number of cases exceeded 3,000,000 in 2021. Notably, within the 30–34 age bracket, the number of cases reached 6,917,889.4 (95%UI: 4,771,275.4–9,269,763.1). The peak incidence rate among women was observed in the 30–34 age group, at 11,832.6 per 100,000 (95%CI: 8,160.3–15,854.0), followed closely by the 25–29 age group with an incidence rate of 11,809.7 (95%CI: 8,284.9–15,991.9). In terms of male incidence, within the age range of 10–59, the number of cases exceeded 3,000,000 in 2021. The highest number of cases among males was recorded in the 30–34 age bracket, at 5,859,058.6 (95%UI: 4,063,551.8–8,073,080.4). Regarding incidence rates, the highest rate among males was found in the 95+ age group, at 10,888.3 (95%CI: 5,714.7–16,725.8), followed by the 30–34 age group with a rate of 9,347.0 (95%CI: 6,482.6, 12,879.1). For migraine (Figure 3B), among women aged 5–59, the number of cases exceeded 400,000 in each age group, with the highest number of cases in the 30–34 age bracket, at 9,545,623.0 (95%UI: 612,390.5–1,389,416.2). The age group with the highest incidence rate was 10–14 years, at 2,257 (95%CI:1,620–2,957), followed by 15–19 years at 1,760.6 (95%CI:1,075.6–2,586.2). Among men, the age groups with more than 400,000 cases were 10–19, 24–29, and 30–39, with the highest number of cases in the 10–14 age group, at 607,431.1 (95%UI: 430,637.5–824,529.3). In terms of incidence rates among men, the 10–19 age bracket had the highest rates, with the 10–14 age group at 1,320.7 (95%CI: 936.3–1,792.7) and the 15–19 age group at 1,082.7 (95%CI: 673.2, 1,594.3). In the case of TTH (Figure 3C), among women, the number of cases exceeded 4,000,000 in the age groups 25 to 54, with the highest number of cases in the 30–34 age bracket, at 5,963,326.5 (95%UI: 3,858,519.7–8,481,019.3). In terms of incidence rates, the highest rate was observed in the 95+ age group, at 11,507.7 (95%CI: 6,185.3–17,642.9), followed by the 30–34 age group with a rate of 10,199.0 (95%CI: 6,599.2–14,505.0). Among men, the number of cases exceeded 400,000 in the 30–39 age bracket, with the highest number in the 30–34 age group, at 5,284,159.0 (95%UI:3,596,444.9–7,569,369.5). The age group with the highest incidence rate among men was the 95+ age group, at 10,862.7 (95%CI: 5,689.3–16,699.9), followed by the 30–34 age group with a rate of 8,429.9 (95%CI: 5,737.4–12,075.5).

#### 4.6.2 Prevalence

In the field of headache disorders, the 2021 statistics reveal that the number of female patients exceeds 12 million across all age groups from 15 to 69. Particularly, the number of patients in the age groups of 30–34, 35–39, 45–49, and 50–54 surpasses 20 million, with the 30–34 age group having the highest number of female patients, reaching 26.5 million (95%UI: 21.3 million-31.6 million). In terms of prevalence rates, the 30–34 age group also ranks first among females, with a prevalence rate of 45,380.4 (95%CI: 36,572.5–54,092.4), followed closely by the 35–39 age group with a prevalence rate of 45,255.7 (95%CI: 36,943.5–54,280.9). For male patients, the number of those affected exceeds 12 million across all age groups from 25 to 59. The 30–34 age group has the highest number of male patients, totaling 21.3 million (95%UI: 17.0 million−26.3 million). In terms of prevalence rates, the 30–34 age group also tops the list among males, with a prevalence rate of 34,054.8 (95%CI: 27,063.7–41,948.1), followed by the 35–39 age group with a prevalence rate of 33,635.2 (95%CI: 26,171.6–41,798.4). In the analysis of migraine, the number of female patients exceeds 8 million across six age groups from 25 to 59. Among them, the 30–34 age group has the largest number of patients, totaling 13.1 million (95%UI: 10.5 million−16.5 million). In terms of prevalence rates, the 40–44 age group has the highest rate, at 23,310.2 (95%CI: 18,283.0-29,166.1), followed by the 35–39 age group with a prevalence rate of 23,189.2 (95%CI: 18,554.0–28,768.8). For male migraine patients, the 30–34 age group has the highest number of patients, totaling 8.4 million (95%UI: 6.6 million−10.6 million). In terms of prevalence rates, the 40–44 age group ranks first among males, with a prevalence rate of 13,672.4(95%CI:10,904.5–17,576.8), closely followed by a similar age group (35–39 ages) with a prevalence rate of 13,650.3 (95%CI: 10,831.6–17,004.2). Regarding tension-type headache (TTH), the number of female patients exceeds 10 million across seven age groups from 25 to 59. Among them, the 30–34 age group has the highest number of patients, totaling 17.3 million (95%UI: 11.7 million−23.7 million). In terms of prevalence rates, the 30–34 age group also has the highest rate among females, at 29,566.7 (95%CI:19,974.5–40,591.7), followed by the 95+ age group with a prevalence rate of 29,340.9 (95%CI:19,208.4–40,851.5). For male TTH patients, the number of those affected exceeds 10 million across five age groups from 30 to 54. Among them, the 30–34 age group has the highest number of patients, totaling 15.0 million (95%UI: 10.3 million-20.6 million). In terms of prevalence rates, the 95+ age group ranks first among males, with a prevalence rate of 27,047.7 (95%CI:17,388.4, 38,456.2), followed by the 30–34 age group with a prevalence rate of 23,859 (95%CI:16,456.8, 32,895.8).

#### 4.6.3 DALYs

In the context of headache disorders, female DALYs across the six age brackets from 30 to 59 years all exceed 300,000, with the 30–34 years age group reporting the highest number at 517,540.8 (95%UI: 68,720.1–1,142,055.4). In terms of DALYs rates, the 40–44 years age group tops the list with 945.8 (95%CI: 169.3–2,121.3), closely followed by the 35–39 years age group with 929.4 (95%CI: 153.6–2,016.0). For males, DALYs are significantly present in the age brackets of 30–39 and 45–54, with the highest number recorded in the 30–34 years age group at 358,049.4 (95%UI: 77,973.0–759,684.5). Regarding DALYs rates, the 40–44 years age group leads with 613.7 (95%CI: 160.5–1,275.5), followed by the 35–39 years age group with 600.8 (95%CI: 153.0–1,255.5). In cases of migraine, female DALYs exceed 400,000 in the age brackets of 30–39 and 45–54, with the 30–34 years age group reporting the highest number at 481,351.5 (95%UI: 45,114.5–1,109,880.1). In terms of DALYs rates, the 40–44 years age group is highest at 874.7 (95%CI: 106.7–2,030.9), followed by the 35–39 years age group with 861.2 (95%CI: 97.4–1,962.6). For male migraine sufferers, the 30–34 years age group reports the highest DALYs number at 322,315.4 (95%UI: 50,577.2–718,138.2). In terms of DALYs rates, the 40–44 years age group leads with 546.6 (95%CI: 114.1–1,193.2), followed by the 35–39 years age group with 536.9 (95%CI:105.4–1,172.3). In cases of TTH, female DALYs in the six age brackets from 30 to 59 years all surpass 300,000, with the highest number reported in the 50–54 years age group at 40,608.3 (95%UI: 11,574.2–108,811.7). In terms of DALYs rates, the 40–44 years age group is highest at 71.1 (95%CI: 21.5–217.4) followed by the 45–49 years age group with 70.3 (95%CI: 20.2–200.1). For males, DALYs in the six age brackets from 30 to 59 years all exceed 300,000, with the highest number recorded in the 50–54 years age group at 38,918.5 (95%UI: 11,958.0–98,951.3). In terms of DALYs rates, the 40–44 years age group leads with 67.1 (95%CI: 21.5–215.8), followed by the 45–49 years age group with 66.1 (95%CI: 20.7–189.2).

### 4.7 Age-period-cohort model for analysis of headache incidence and prediction of changes in future disease burden

**Figure 4:**
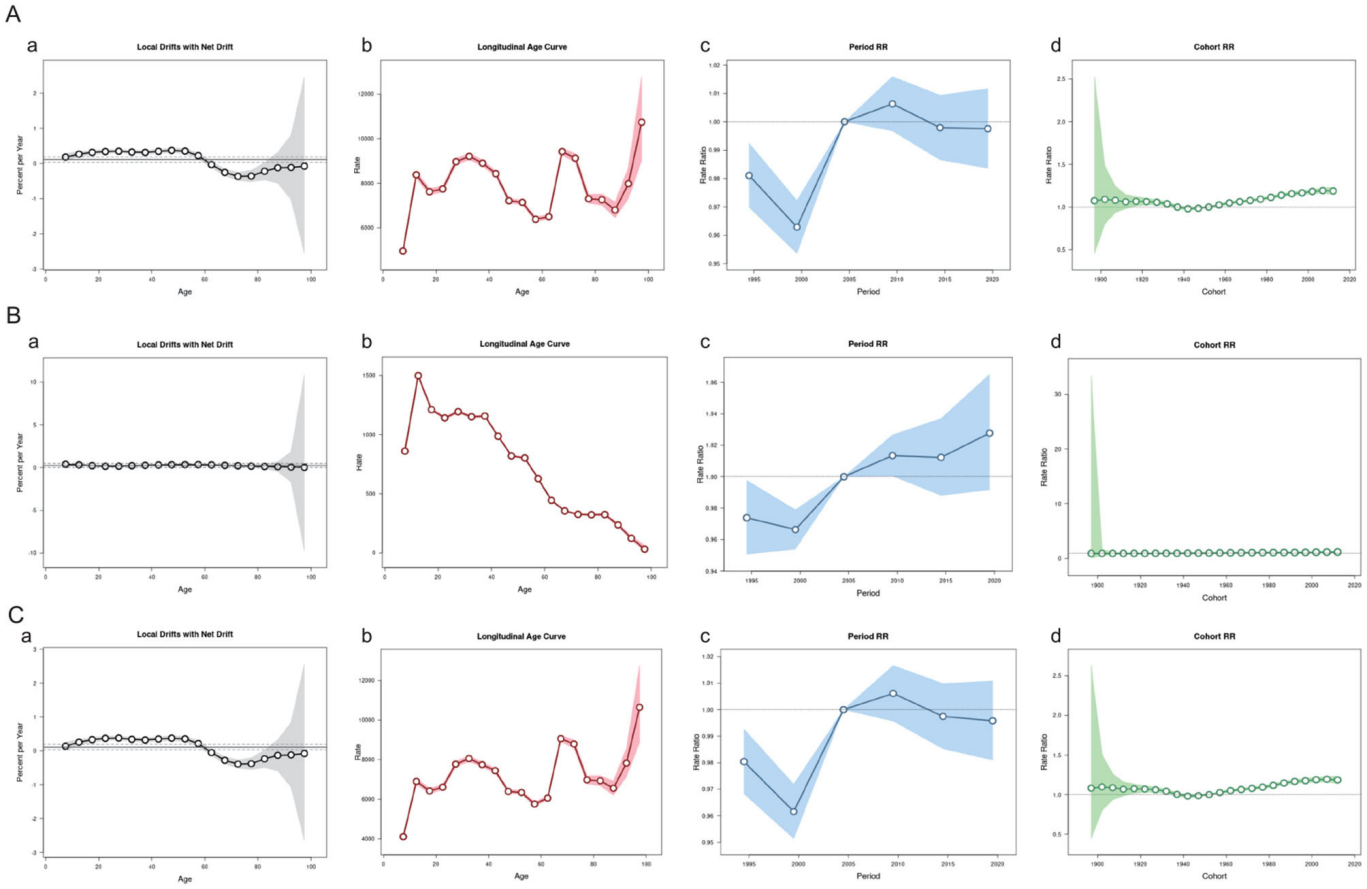
Age-Period-Cohort Model of the headache disorders, migraine and TTH in China. **(A)** Age-Period-Cohort Model about the Headache disorders. **(B)** Age-Period-Cohort Model about Migraine. **(C)** Age-Period-Cohort Model about TTH.

Firstly (Figure 4A), examining the overall trend of headache disorders, the annual Net Drift is 0.1125%, with a *p* < 0.01, indicating a statistically significant upward trend in the incidence of headache disorders. Data on Local Drifts reveal that the incidence increases year by year up to the age of 57.5, after which it begins to decline around the age of 60. In terms of age effects, the incidence is higher than the overall average in the age groups of 12.5–42.5 years and 67.5–72.5 years. The incidence of headache disorders peaks significantly around the age of 10, remains relatively stable but at a high level between the ages of 20 and 40, and then gradually decreases before 60 years of age. However, there is a notable increase in incidence around the age of 87.

In the analysis of the Rate Ratio (RR) across different periods, with May 2004 as the reference point, there was a significant increase in the incidence of headache disorders from 2000 to 2010. After 2010, the RR decreased but remained above 1, suggesting that the incidence continued to be higher than the baseline level. Since 2015, the RR has stabilized. In terms of changes in RR across birth cohorts, there was a slight increase in risk for those born between 1952 and 2012, but it generally remained around 1, indicating no significant change in incidence between cohorts.

For migraine (Figure 4B), the APC model shows an annual Net Drift of 0.2411%, which is statistically significant (*p* < 0.05). Although the average trend of change across all age groups is not pronounced, the overall incidence of migraine is increasing. Among the age groups from 5 to over 95 years, the Percent per Year decreased from 0.378% to 0.0321%. The incidence of migraine peaks around the age of 12, remains at a higher level between the ages of 17.5 and 37.5, and then gradually decreases. In the changes of RR across different periods, the RR was below the baseline value of 1 from 1995 to 2005, after which it gradually increased, indicating a continuous rise in the incidence of migraine in recent years. In the changes of RR across birth cohorts, the RR for those born between 1952 and 2012 was slightly above 1, but the variation was minimal, suggesting no significant change in incidence between cohorts.

Lastly (Figure 4C), the APC trend analysis for tension-type headache shows an annual Net Drift of 0.1108%, which is statistically significant. The annual change in incidence is relatively small before the age of 60, indicating stability. After the age of 60, the incidence decreases significantly. The incidence of tension-type headache is relatively high at various ages, such as 12.5, 32.5, and 67.5 years old. In the changes of RR across different periods, there was a significant decrease in incidence from 1995 to 2000, followed by a gradual increase, surpassing the baseline value of 1 after 2005, peaking between 2009 and 2010, and then decreasing. In the changes of RR across birth cohorts, the RR for those born between 1920 and 2012 has been stable, indicating no significant fluctuation in the incidence of tension-type headache between cohorts over these years.

### 4.8 The ARIMA model predicts the incidence of different sex in china

**Figure 5:**
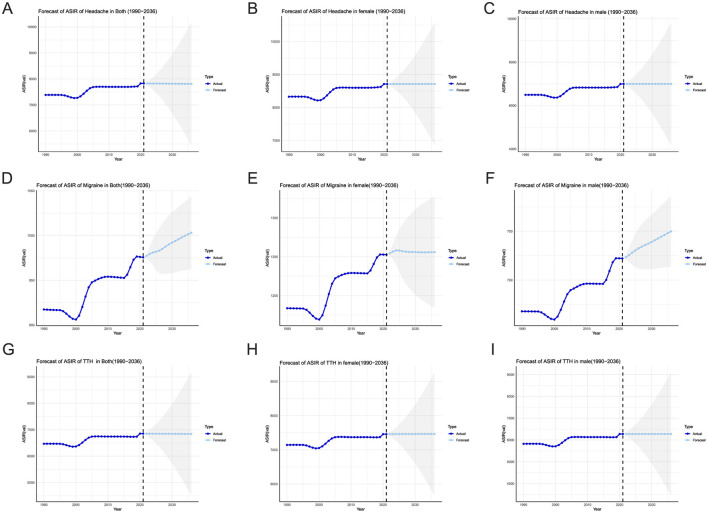
ARIMA model predicts the incidence of the headache disorders in China, gray shaded part is the confidence interval. **(A–C)** He ARIMA model predicted AISA the headache disorders about both, female, and male. **(D–F)** The ARIMA model predicted AISA of the migraine about both, female, and male. **(G–I)** The ARIMA model predicted AISA of the TTH about both, female, and male.

We utilized the data from GBD 2021 database to forecast the ASIR of Headache disorders, in China over the next 5 years. The detailed prediction results are presented in Supplementary Table S3 and Figure 5. In terms of both incidence rate of headache, the projections indicate a stable trend, with a slight decline from 7,826.7 in 2021 to 7,824.2 in 2036. The incidence rate among female patients remains stable, maintaining a consistent level of 8,712.7 in 2021 and 8,713.1 in 2036, with not obviously change in their ASIR data. The predicted incidence rate for male patients aligns with the 2021 ASIR data, stabilizing at 6,996.7. According to the projections, the incidence rate among female patients will continue to be higher than that among male patients over the next 15 years, with no apparent trends of decrease or improvement.

Regarding migraines (Figures 5D–F), the ASIR data shows an increase from 975.6 in 2021 to 1,003.2 in 2036, suggesting a growing trend in incidence rates. Specifically, although the incidence rate among female patients increases from 1,252.7 in 2021 to 1,256.0 in 2036, the increase is relatively modest. In contrast, the ASIR among male patients increases more significantly, from 722.2 in 2021 to 749.8 in 2036.

Additionally, we have also predicted the ASIR of TTH over the next 15 years based on the ASIR data from the past 30 years (Figures 5G–I). The results indicate that the incidence rate for the general population will remain stable, with a slight decline from 6,851.1 in 2021 to 6,849.8 in 2036. The incidence rate among female patients remains stable as well, ranging from 7,460.0 in 2021 to 7,460.6 in 2036. The incidence rate among male patients remains consistent from 6,274.5 to 6,274.7 throughout the prediction period, showing no significant changes.

## 5 Discussion

Headache disorders, particularly migraines and TTH, have long-term, recurring characteristics and may be accompanied by lifelong characteristics. Often, only by effectively reducing the incidence of head pain and continuously optimizing treatment strategies can the burden of this type of disease be effectively reduced (14).

This study analyzed data from GBD 2021 and found that the incidence, prevalence, and DALYs of headache disorders in China are higher than the global average. Since 1990, the standardized prevalence rates of migraine and tension-type headache in China have shown an upward trend, with increases of 7.6% and 7.9%, respectively. In contrast, China's Age-Standardized Death Rate (ASDR) related to these conditions has remained relatively stable, ranging from 454.8 in 1990 to 487.2 in 2021. This stability may be attributed to significant improvements in China's economy and healthcare standards, as well as the sophistication of clinical experience and diagnostic and treatment techniques among neurologists and neurosurgeons. Joinpoint regression analysis revealed that over the past three decades, China has experienced a significant upward trend in the ASIR, AAPC: 0.1930 [0.1537, 0.2324], ASPR, AAPC: 0.2208 [0.1793, 0.2623], and ASDR, AAPC: 0.234 [0.1867, 0.2814] of headache disorders, highlighting China as one of the region's most severely affected by the global burden of headache diseases (15). The age-standardized rates (ASRs) related to headaches worldwide have shown a stable trend, a change closely associated with the global campaign against headache disorders jointly initiated by the World Health Organization (WHO) and the International Headache Society (IHS), among others (16). These discoveries further emphasize the importance of conducting in-depth research and implementing effective prevention and control measures for headaches, particularly focusing on their prevalence trends in specific age groups and genders.

In the gender and age analysis in China, we found that ASIR and ASDR of headache disorders were higher in women than in men. This finding aligns with previous research, which indicated that DALYs due to headaches rank second only to gynecological diseases among young women, and tenth among young men (17). This disparity may be closely linked to female sex hormones and genetic factors. For instance, women experience significant hormonal fluctuations during menstruation, pregnancy, and menopause (18, 19). These changes may increase the sensitivity of the trigeminovascular system by altering brain network activation, leading to the release of inflammatory markers, and ultimately triggering migraines (20). Furthermore, the transformation of China's socioeconomic structure has, to some extent, contributed to the onset and progression of headache disorders. As women take on increasingly significant roles in both family and society (21), the burden of managing household responsibilities and professional pressures often exposes them to heightened psychological and physiological stress. This may increase their sensitivity to pressure and pain while reducing pain tolerance, thereby promoting the development and progression of tension-type headaches (10).

The incidence and disease burden of headache disorders are most prominent among young and middle-aged adults, particularly peaking in the 30–34 age group. This demographic is at a critical stage of building careers and families, balancing responsibilities such as raising children and maintaining household finances while facing pressures from frontline work and complex social relationships in their professional lives. The dual burden of family and societal pressures often leads to fatigue and anxiety (22, 23) and may even contribute to chronic inflammation or suboptimal health, thereby increasing the risk of headache disorders (24, 25). Additionally, the burden of headache disorders shows a transient rise in the 10–14 age group (Figures 3A–C), which may be closely linked to initial hormonal changes during puberty (26). Meanwhile, shifts in leisure activities, excessive use of electronic devices, and sleep deprivation may also trigger or exacerbate headache disorders in adolescents (27, 28).

ARIMA model projections suggest if there are no effective public health interventions or public health emergencies, based on the incidence of different genders in China over the past 30 years and found that the incidence of women in the next 15 years remained stable that in 2021. The incidence of men is expected to increase in the migraine over the next 15 years. Unhealthy lifestyle habits among men (29), such as smoking, alcohol consumption, and staying up late, can negatively impact the neurovascular system, potentially triggering or exacerbating migraines. Additionally, within China's social structure, men are typically more engaged in physically demanding labor, and intense physical activity has been identified as a potential trigger for migraine onset and worsening (30). The heightened sensitivity to exercise in migraine patients may stem from abnormal activation of nociceptive fibers (31). Furthermore, inadequate nutritional intake, dehydration, and prolonged exposure to intense sunlight are widely recognized as common triggers for migraines (32–34). Notably, as the incidence and disease burden of headaches are significantly higher in women than in men, current medical strategies and treatment resources may be disproportionately focused on women, which could contribute to a projected increase in the age-standardized incidence rate (ASIR) of migraines among men over the next 15 years.

This study has several limitations. First, although the GBD study integrates a wide range of data sources, data scarcity in certain regions or populations may affect the accuracy of disease burden estimates. Second, headaches are often misdiagnosed as other conditions (e.g., stroke, cervical spondylosis, or anxiety disorders), potentially leading to an underestimation of headache incidence in the GBD; conversely, if non-headache conditions (e.g., sinusitis or temporomandibular joint disorders) are misdiagnosed as headaches, the incidence may be overestimated. Additionally, ARIMA-based predictions have limited capacity to account for sudden public health events and non-linear changes, which may compromise the reliability of the results. Given these limitations, future research will further evaluate the burden of headache disorders in China by incorporating additional data sources (e.g., provincial-level data) and refining modeling approaches.

## 6 Conclusion

Headache disorders, long overlooked and recurrent, impose a substantial health and economic burden on China, particularly among women. To address this challenge, this study proposes a multi-faceted intervention strategy: enhancing public education to raise awareness, optimizing healthcare resource allocation to strengthen primary care physicians' diagnostic capabilities, and promoting “cloud clinic” telemedicine in underserved areas. Additionally, improving social security policies to increase outpatient reimbursement rates can alleviate financial burdens, while fostering international collaboration to advance the development of novel therapeutics. These measures are expected to significantly reduce the societal burden of headache disorders, elevate prevention and treatment standards, and provide a scientific foundation for public health policy formulation.

## Data Availability

The original contributions presented in the study are included in the article/Supplementary material, further inquiries can be directed to the corresponding author.
