# Supplementary material for: Analysis of headache burden Chinese in the global context from 1990 to 2021
Source: Front Neurol. 2025 Apr 16;16:1559028. doi: 10.3389/fneur.2025.1559028 (PMC12040657; doi:10.3389/fneur.2025.1559028)
Supplement: Supplementary file 1 [file Supplementary_file_1.zip › supplementary tables:figures/Supplementary table.docx]

**TableS1- The comparison of the migraine and TTH burden data in China and global in 1990 and 2021.**

| **Location** | | **Category** | **Measure** | | **1990** | | **2021** | |
| --- | --- | --- | --- | --- | --- | --- | --- | --- |
|  |  | | | **Number** | | **Rate** | **Number** | **Rate** |
|  |  | | | | **n(95%UI)** | **n(95%UI)** | **n(95%****UI)** | **n(95%UI)** |
| **China** | | **Migraine** | **Incidence** | | 11518097.6(10091942.2, 13156841.9) | 979.0(857.8, 1118.3) | 13047220.7 (11597731.5, 14698852.1) | 917.0 (815.2, 1033.1) |
|  |  |  | **Prevalence** | | 133474537.5(114199443.7, 153482597.7) | 11345.5 (9707.1, 13046.2) | 184752280.1(160836524.7, 213633958.3) | 12985.6 (11304.7, 15015.6) |
|  |  |  | **DALYs** | | 5028787.5(767668.5, 11262271.5) | 427.5(65.3, 957.3) | 6988198.6 (15186289.3,1133318.7) | 491.2 (79.7, 1067.4) |
|  |  | **TTH** | **Incidence** | | 76930406.5(66380713.5, 87690508.6) | 6539.2 (5642.4,7453.8) | 102020342.6(88865128.1, 115000579.6) | 7170.7(6246.0, 8083.0) |
|  |  |  | **Prevalence** | | 204064313.2(176898604.5, 233568232.2) | 17345.7 (15036.5, 19853.5) | 283814151.0(251438661.8, 320431556.8) | 19948.3 (17672.8, 22522.0) |
|  |  |  | **DALYs** | | 489462.7 (151736.8, 1687075.4) | 41.6(12.9,143.4) | 716164.9 (224403.1, 2174717.2) | 50.3(15.8, 152.9) |
| **Global** | | **Migraine** | **Incidence** | | 63496590.8 (55194751.5, 72208003.4) | 1190.5 (1034.8,1353.8) | 90183386.9 (78857600.5, 101838162.5) | 1142.8 (999.3, 1290.5) |
|  |  |  | **Prevalence** | | 732564462.7(624559243.9, 847058436.3) | 13734.8 (11709.8,15881.5) | 1158432823.8 (995861966.4, 1331312506.1) | 14679.8(12619.7, 16870.5) |
|  |  |  | **DALYs** | | 27412196.3 (4076605.0, 60325805.8) | 514.0 (76.4, 1131.0) | 43378889.8(6732642.2, 95079454.1) | 549.7(85.3, 1204.9) |
|  |  | **TTH** | **Incidence** | | 470298233.1(408471892.0, 527847536.3) | 8817.6 (7658.4, 9896.6) | 719043093.3(629219079.9, 804949048.8) | 9111.8 (7973.5, 10200.4) |
|  |  |  | **Prevalence** | | 1286366671.7(1122503420.8, 1467160187.5) | 24118.1 (21045.8, 27507.8) | 2011612877.5(1776544390.8, 2270860638.4) | 25491.4 (22512.5, 28776.6) |
|  |  |  | **DALYs** | | 2848687.6 (820890.8, 9563745.4) | 53.4 (15.4, 179.3) | 4596785.3 (1347300.8, 15012932.8) | 58.3 (17.1, 190.2) |

**TableS2-Comparison of the burden of Headache disorders, Migraine and TTH in Chinese men and women in 1990 and 2021**

| **Sex** | | **Category** | **Measure** | | **1990** | | **2021** | |
| --- | --- | --- | --- | --- | --- | --- | --- | --- |
|  |  | | | **Number** | | **Rate** | **Number** | **Rate** |
|  |  | | | | **n(95%UI)** | **n(95%UI)** | **n(95%UI)** | **n(95%UI)** |
| **Female** | | **Headache disorders** | **Incidence** | | 48524705.3(42751091.2, 54287780.8) | 8518.8(7505.2,9530.6) | 62789137.9(55836988.0, 69851764.2) | 9039.1(8038.2, 10055.8) |
|  |  |  | **Prevalence** | | 173941124.8(159508023.1, 189576813.5) | 30536.4(28002.6, 33281.4) | 239919680.9(222532359.8, 258926544.2) | 34538.6(32035.5, 37274.8) |
|  |  |  | **DALYs** | | 3290317.8(523750.2, 7158034.1) | 577.6(91.9,1256.6) | 4610137.1(809056.8, 9786313.9) | 664(116,1409) |
|  |  | **Migraine** | **Incidence** | | 7176146.2 (6275054.9, 8198406.5) | 1259.8 (1101.6, 1439.3) | 8081262.4 (7209928.8, 9102490.1) | 1163.4 (1037.9, 1310.4) |
|  |  |  | **Prevalence** | | 82587175.2(70478899.5, 94673001.5) | 14498.7 (12373.0,16620.4) | 114716057.2(98856230.6, 132076637.6) | 16514.4 (14231.2, 19013.6) |
|  |  |  | **DALYs** | | 3041524.2(360236.2, 6866451.0) | 534.0(63.2, 1205.4) | 4242877.9 (548619.6, 9335915.4) | 610.8 (79.0, 1344.0) |
|  |  | **TTH** | **Incidence** | | 41348559.0(35655137.9, 46914757.2) | 7259.0 (6259.5, 8236.2) | 54707875.4 (47888419.2, 61745536.1) | 7875.7(6894.0, 8888.8) |
|  |  |  | **Prevalence** | | 110367560.8(95575144.3, 126490494.4) | 19375.7(16778.8, 22206.2) | 153608982.8(135965647.4, 173917944.9) | 22113.4 (19573.5, 25037.0) |
|  |  |  | **DALYs** | | 248793.6(72186.7, 842240.2) | 43.7(12.7,147.7) | 367259.2 (108105.6, 1130637.9) | 52.9(15.6, 162.8) |
| **Male** | | **Headache disorders** | **Incidence** | | 39923798.8(34856399.9, 45042919.2) | 6579.0(5743.9,7422.5) | 52278424.4(45790801.0, 58481324.4) | 7180.1(6289.1,8032.0) |
|  |  |  | **Prevalence** | | 135256759.5(123112446.3, 148349280.5) | 22288.7(20287.5, 24446.2) | 186092867.1(169878674.3, 203555354.3) | 25558.6(23331.7, 27956.9) |
|  |  |  | **DALYs** | | 2227932.4(562076.1, 4623760.7) | 367.1(92.6,761.9) | 3094226.5(832974.8,6274830.6) | 425.0(114.4,861.8) |
|  |  | **Migraine** | **Incidence** | | 4341951.3(3779869.6, 4957833.5) | 715.5(622.9,817.0) | 4965958.2 (4347456.2, 5638304.9) | 682.0 (597.1, 774.4) |
|  |  |  | **Prevalence** | | 50887361.4(43387428.2, 59129685.5) | 8385.6 (7149.7,9743.9) | 70036223.0(60652046.1, 81324228.2) | 9619.0(8330.1, 11169.3) |
|  |  |  | **DALYs** | | 1987263.3(394504.7, 4365126.3) | 327.5 (65.0, 719.3) | 2745320.7(581676.9, 5851443.2) | 377.1(79.9, 803.7) |
|  |  | **TTH** | **Incidence** | | 35581847.5(30530734.4, 40778919.4) | 5863.5 (5031.1, 6719.9) | 47312466.1(40984744.0, 53242664.9) | 6498.0 (5629.0, 7312.5) |
|  |  |  | **Prevalence** | | 93696752.4(81301112.7,107220269.2) | 15440.1 (13397.5,17668.6) | 130205168.2(113609949.8,148215066.5) | 17882.8(15603.5, 20356.3) |
|  |  |  | **DALYs** | | 240669.1 (78456.8, 873200.8) | 39.7 (12.9, 143.9) | 348905.8 (115344.2, 1113502.0) | 47.9 (15.8, 152.9) |

**TableS3-Predicting incidence data 15 years later based on ASIA since 1990 in China**

|  | **Rate of Incidence** | | | | | | | | | | | |
| --- | --- | --- | --- | --- | --- | --- | --- | --- | --- | --- | --- | --- |
|  | **Sex** | **Actual** | | | | | | | **Forecast** | | |  |
| **Classification** |  | **1991** | **1996** | **2001** | **2006** | **2011** | **2016** | **2021** | **2026** | **2031** | **2036** |  |
| **Headache disorders** | **Both** | 7386.1 | 7366.3 | 7330.3 | 7696.3 | 7694.8 | 7693.5 | 7826.7 | 7824.5 | 7824.3 | 7824.2 |  |
|  | **Female** | 8329.5 | 8311.4 | 8277.3 | 8603.2 | 8599.6 | 8602.0 | 8712.7 | 8713.0 | 8713.1 | 8713.1 |  |
|  | **Male** | 6494.0 | 6474.0 | 6430.0 | 6828.3 | 6828.2 | 6829.7 | 6996.7 | 6996.7 | 6996.7 | 6996.7 |  |
| **Migraine** | **Both** | 917.3 | 915.5 | 910.4 | 949.6 | 953.8 | 956.2 | 975.6 | 983.2 | 994.0 | 1003.2 |  |
|  | **Female** | 1183.7 | 1181.8 | 1174.7 | 1224.6 | 1229.1 | 1232.4 | 1252.7 | 1257.2 | 1255.9 | 1256.0 |  |
|  | **Male** | 668.0 | 666.9 | 662.7 | 691.4 | 696.3 | 700.1 | 722.2 | 732.5 | 740.8 | 749.8 |  |
| **TTH** | **Both** | 6469.0 | 6450.8 | 6419.9 | 6746.6 | 6741.0 | 6737.3 | 6851.1 | 6849.9 | 6849.8 | 6849.8 |  |
|  | **Female** | 7145.8 | 7129.6 | 7102.6 | 7378.6 | 7370.5 | 7369.6 | 7460.0 | 7460.5 | 7460.6 | 7460.6 |  |
|  | **Male** | 5826.0 | 5807.1 | 5767.4 | 6137.0 | 6131.9 | 6129.6 | 6274.5 | 6274.7 | 6274.7 | 6274.7 |  |
