# Supplementary material for: Analysis of headache burden Chinese in the global context from 1990 to 2021
Source: Front Neurol. 2025 Apr 16;16:1559028. doi: 10.3389/fneur.2025.1559028 (PMC12040657; doi:10.3389/fneur.2025.1559028)
Supplement: Supplementary file 1 [file Supplementary_file_1.zip › supplementary tables:figures/Fig.S1.pdf]

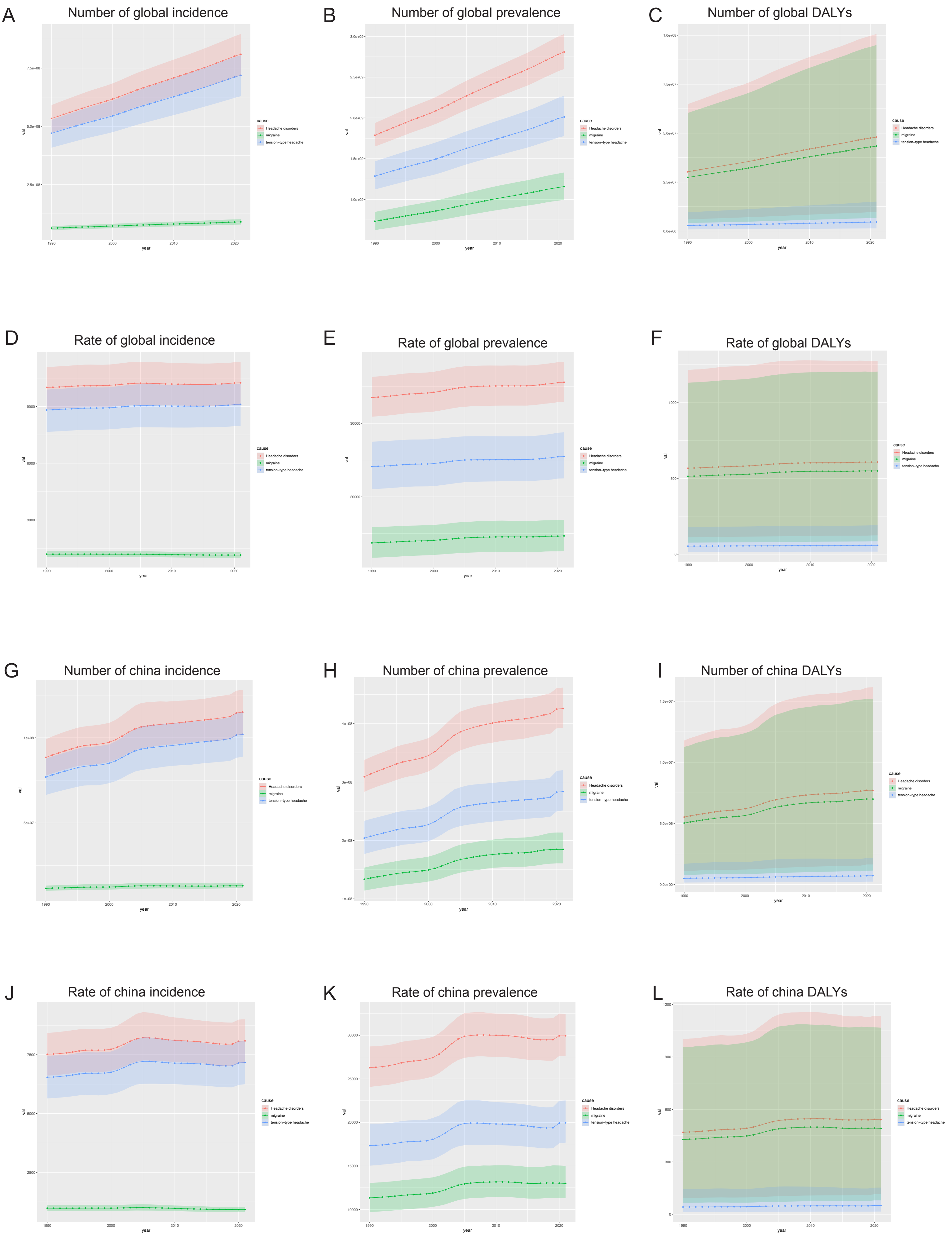

A
Number of global incidence
B
Number of global prevalence
C
Number of global DALYs
D
Rate of global incidence
E
Rate of global prevalence
F
Rate of global DALYs
G
Number of china incidence
H
Number of china prevalence
I
Number of china DALYs
J
Rate of china incidence
K
Rate of china prevalence
L
Rate of china DALYs
cause
Headache disorders
migraine
tension−type headache
val
year
1990
2000
2010
2020
