# Supplementary figures and images for: Analysis of headache burden Chinese in the global context from 1990 to 2021
Source: Front Neurol. 2025 Apr 16;16:1559028. doi: 10.3389/fneur.2025.1559028 (PMC12040657; doi:10.3389/fneur.2025.1559028)

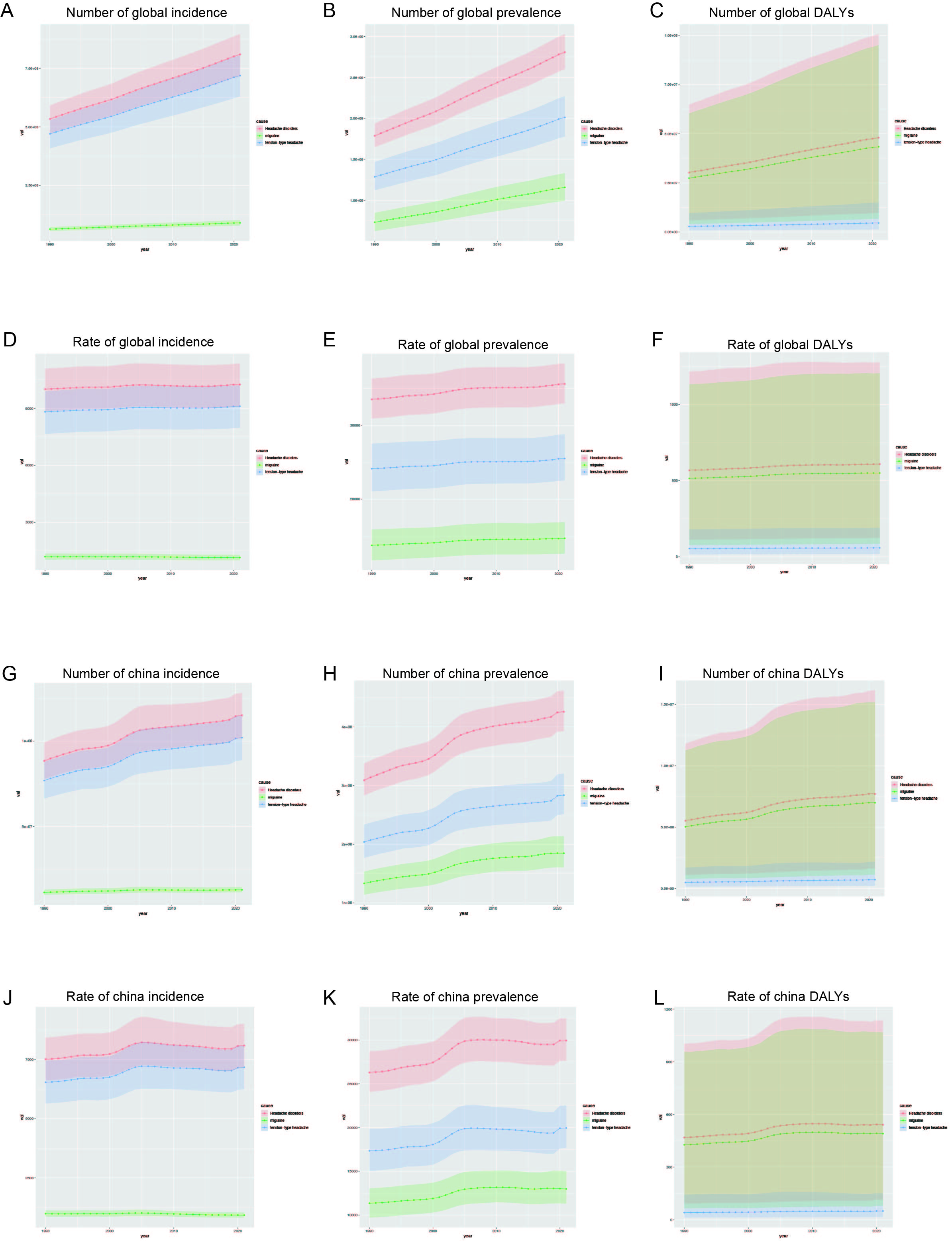

Supplement: Supplementary file 1 [file Supplementary_file_1.zip › supplementary tables:figures/Fig.S1.jpg]

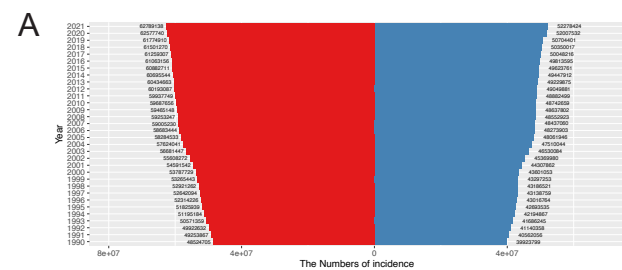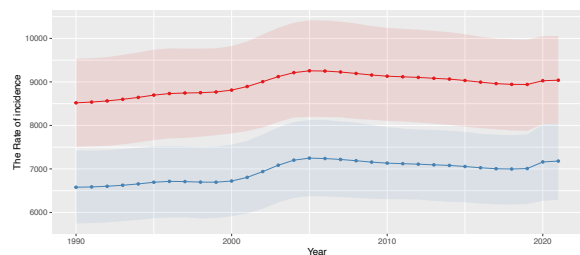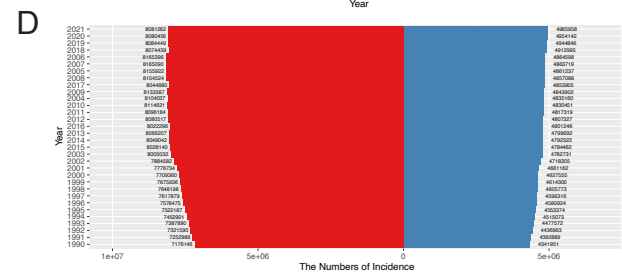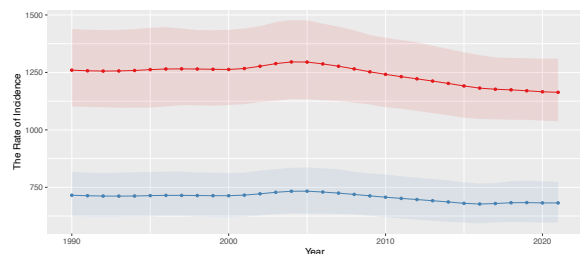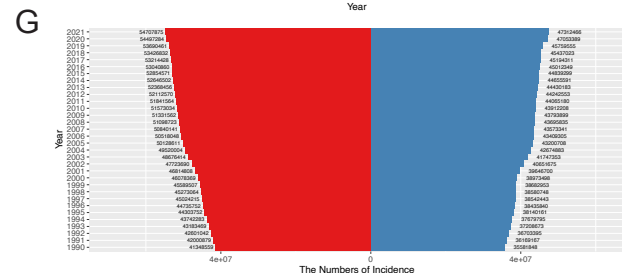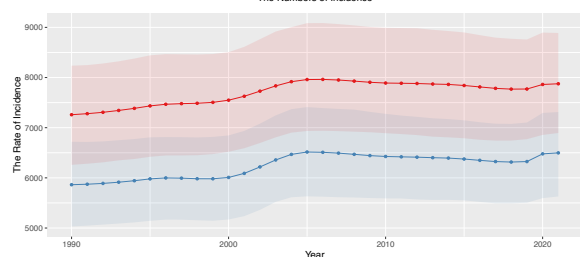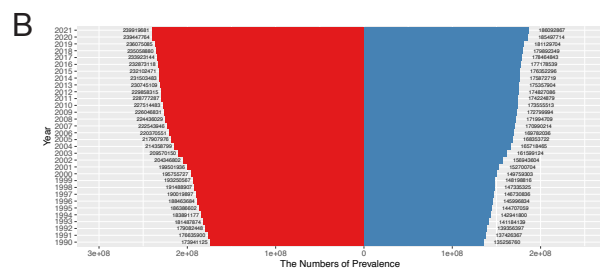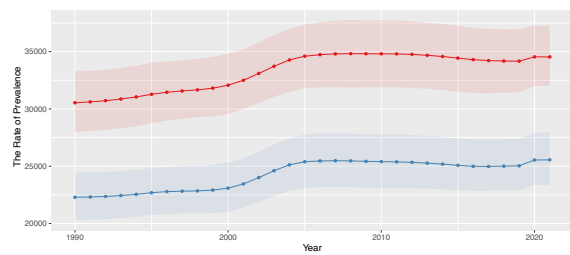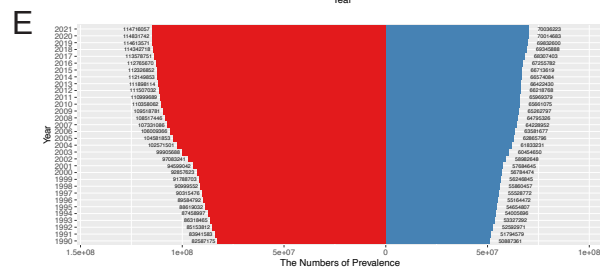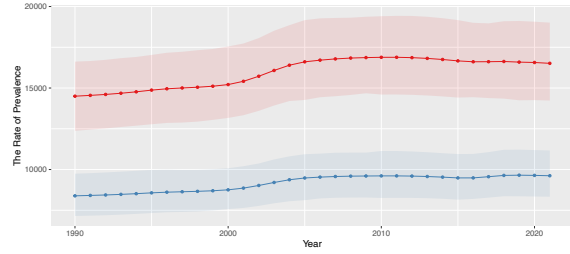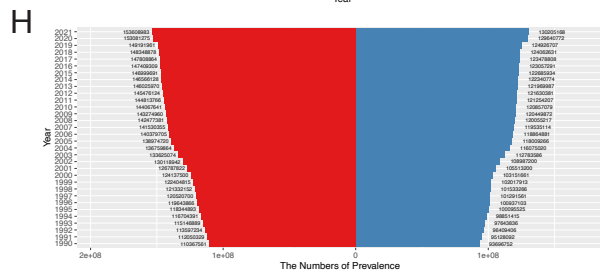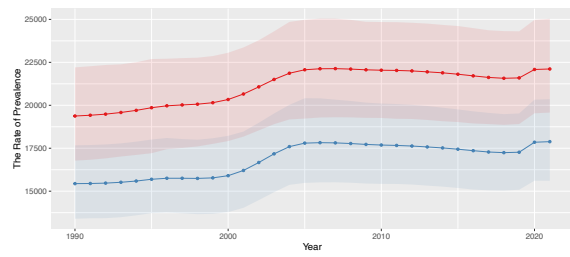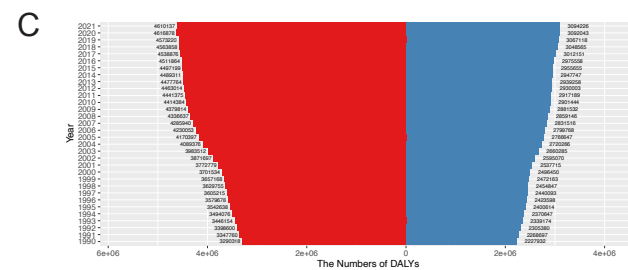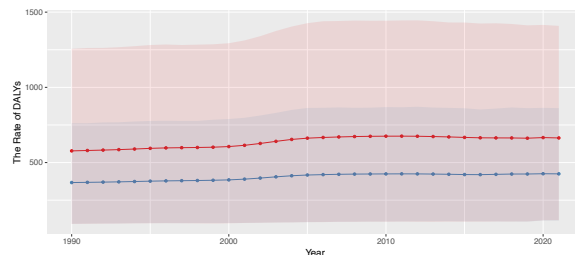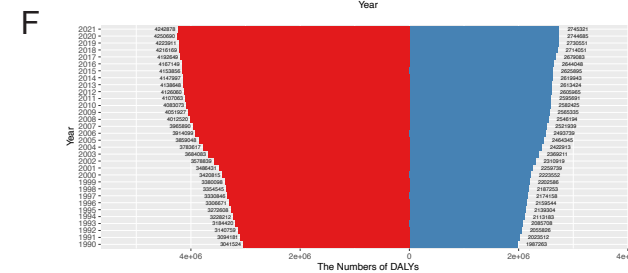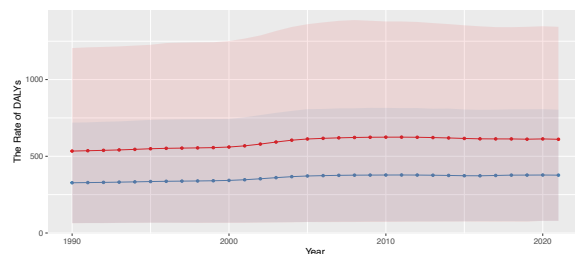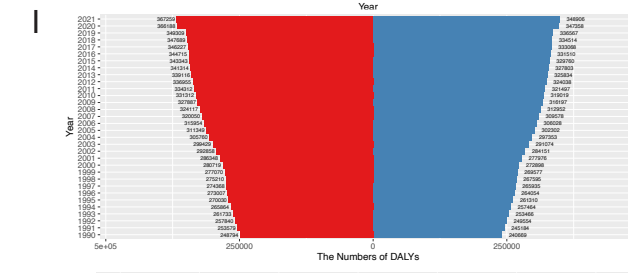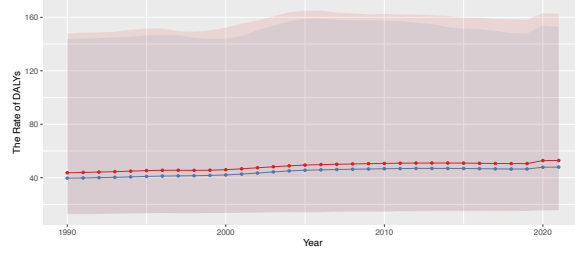

Supplement: Supplementary file 1 [file Supplementary_file_1.zip › supplementary tables:figures/Fig.S2.pdf]

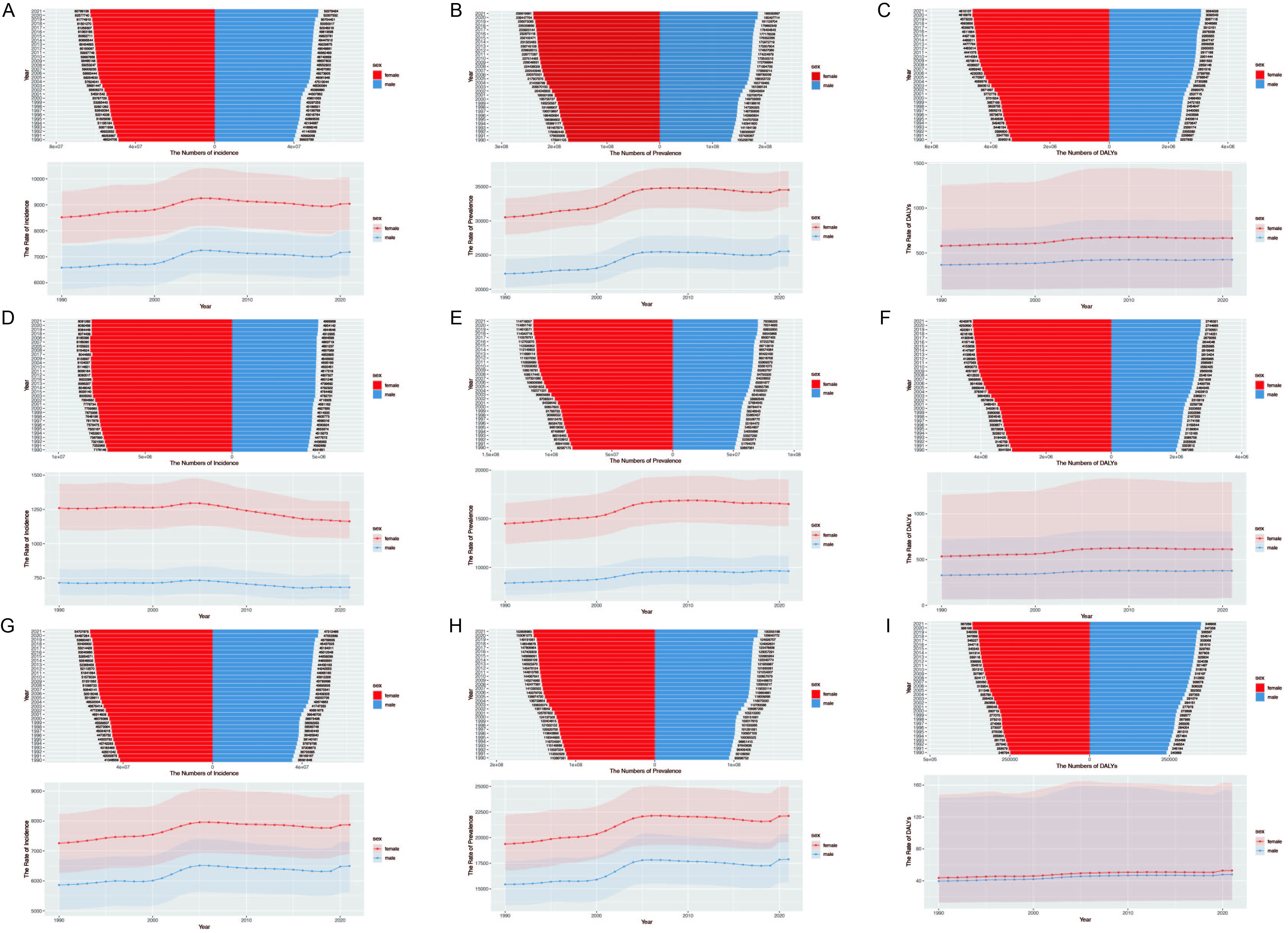

Supplement: Supplementary file 1 [file Supplementary_file_1.zip › supplementary tables:figures/Fig.S2.jpg]
